# Regulation of tyrosine hydroxylase is preserved across different homo- and heterodimeric 14-3-3 proteins

**DOI:** 10.1007/s00726-015-2157-0

**Published:** 2016-01-29

**Authors:** Sadaf Ghorbani, Agnete Fossbakk, Ana Jorge-Finnigan, Marte I. Flydal, Jan Haavik, Rune Kleppe

**Affiliations:** Department of Biomedicine, University of Bergen, Bergen, Norway; K.G. Jebsen Centre for Neuropsychiatric Disorders, University of Bergen, Bergen, Norway; Haukeland University Hospital, Bergen, Norway

**Keywords:** 14-3-3, Heterodimer, Isoform, Tyrosine hydroxylase, Activation

## Abstract

Tyrosine hydroxylase (TH) is regulated by members of the 14-3-3 protein family. However, knowledge about the variation between 14-3-3 proteins in their regulation of TH is still limited. We examined the binding, effects on activation and dephosphorylation kinetics of tyrosine hydroxylase (TH) by abundant midbrain 14-3-3 proteins (β, η, ζ, γ and ε) of different dimer composition. All 14-3-3 homodimers and their respective 14-3-3ε-heterodimers bound with similar high affinity (*K*_d_ values of 1.4–3.8 nM) to serine19 phosphorylated human TH (TH-pS19). We similarly observed a consistent activation of bovine (3.3- to 4.4-fold) and human TH-pS19 (1.3–1.6 fold) across all the different 14-3-3 dimer species, with homodimeric 14-3-3γ being the strongest activator. Both hetero- and homodimers of 14-3-3 strongly inhibited dephosphorylation of TH-pS19, and we speculate if this is an important homeostatic mechanism of 14-3-3 target-protein regulation in vivo. We conclude that TH is a robust interaction partner of different 14-3-3 dimer types with moderate variability between the 14-3-3 dimers on their regulation of TH.

**Electronic supplementary material:**

The online version of this article (doi:10.1007/s00726-015-2157-0) contains supplementary material, which is available to authorized users.

## Introduction

Tyrosine hydroxylase (TH) is the first and rate-limiting enzyme in the biosynthesis of catecholamines (Nagatsu et al. 1964), important hormones and neuromodulators. The cellular activity of TH is regulated through several signaling pathways that target TH by Ser/Thr phosphorylation (Almas et al. 1992; Haycock et al. 1982; Haycock and Wakade 1992; Sutherland et al. 1993), and by protein–protein interactions with 14-3-3 proteins (Itagaki et al. 1999; Toska et al. 2002; Yamauchi et al. 1981). Whereas Ser40 phosphorylation potently activates TH (Almas et al. 1992; Andersson et al. 1992; Sura et al. 2004), activation of TH by Ser19 phosphorylation (TH-pS19) requires 14-3-3 binding (Itagaki et al. 1999; Toska et al. 2002; Yamauchi et al. 1981), although this activation of TH has been questioned by other studies (Haycock and Wakade 1992; Sutherland et al. 1993).

The 14-3-3 proteins are highly conserved throughout evolution and are encoded by multiple genes in all eukaryotic species. Whereas yeast has two genes encoding 14-3-3 proteins, plants and mammals have numerous genes (13 and 7 in Arabidopsis and mammals, respectively) coding for different 14-3-3 isoforms (Aitken 2006; Paul et al. 2012). The mammalian 14-3-3s, except 14-3-3σ, are abundantly expressed in the brain, with genes *Ywha(b/g/e/h/s/q* and *z)* corresponding to 14-3-3 proteins (β/γ/ε/η/σ/τ and ζ), respectively (Ichimura et al. 1988). The different monomers can form homo- or heterodimers, and 14-3-3ε preferentially and spontaneously forms heterodimers with the other isoforms (Chaudhri et al. 2003; Jones et al. 1995; Yang et al. 2006).

The notion that the various 14-3-3 proteins can combine into homo- and heterodimeric proteins has caused uncertainty concerning the functional diversity across the different 14-3-3 isoforms in terms of target binding specificity. This issue remains unresolved, particularly as binding to different 14-3-3 dimer combinations has been compared only for few target proteins. In particular, a quantitative comparison of binding affinity for different 14-3-3 homo- and heterodimer types is not available.

Several 14-3-3 proteins play important roles in the mature nervous system, as functional knockout (KO) mice show distinct morphological and behavioral abnormalities (Foote et al. 2015), whereas the 14-3-3ε isoform plays the most prominent role in neural development (Toyo-oka et al. 2003). As 14-3-3ε preferentially forms heterodimers with other 14-3-3 isoforms (except 14-3-3σ) (Yang et al. 2006), this could suggest a particular functional importance of the heterodimeric 14-3-3s. KO studies of several 14-3-3 genes give abnormalities related to major neuropsychiatric disorders, consistent with aberrant expression and association of several 14-3-3 isoforms with such disorders (Jacobsen et al. 2015; Rivero et al. 2015). Thus, various effects on dopamine-/noradrenergic neurotransmission have been reported. The KO model of 14-3-3ζ shows hyperactivity, possibly by altered regulation of the dopamine transporter (Ramshaw et al. 2013), and the heterozygous 14-3-3ε KO mice have an impaired development of tyrosine hydroxylase (TH) positive nerve terminals (Sekiguchi et al. 2011). Thus, the different 14-3-3 proteins may regulate monoamine transmission and biosynthesis to variable degrees.

In vitro and cell studies suggest differences between the 14-3-3 isoforms in their regulation of TH (Halskau et al. 2009; Nakashima et al. 2007; Wang et al. 2009). We therefore wanted to study different isoforms of 14-3-3 in their binding and regulation of TH. In particular, we wanted to include the respective 14-3-3ε-heterodimers. Such 14-3-3 heterodimers have been reported to display strong target specificity (Liang et al. 2008; Rajagopalan et al. 2010) and the 14-3-3ε KO mice show high lethality and dramatic neurodevelopmental defects (Toyo-oka et al. 2003).

Using purified recombinant proteins as well as TH from native source, we performed a comparative study of the different hetero- and homodimers of mammalian 14-3-3 isoforms that are reportedly expressed in dopaminergic midbrain neurons (β, γ, Ɛ, η, and ζ) (Wang et al. 2009). We measured the binding affinity of different 14-3-3 dimers to TH phosphorylated on Ser19, their effects on TH activation, as well as their effect on the dephosphorylation of TH-pS19.

## Materials and methods

### Materials

[^32^P-γ]ATP was from PerkinElmer (Georgia, USA). (6R)-Tetrahydrobiopterin (BH_4_) and 6-methyl-tetrahydrobiopterin (6MPH_4_) was from Dr B. Schirck’s Laboratories (Jona, Switzerland). pGEX-2T plasmids for expression of 14-3-3γ, η, and ζ were gifts from Prof. Alastair Aitken (Edinburgh, UK), for 14-3-3β from Dr. C. James Hastie [MRC, Division of Signal Transduction Therapy, University of Dundee (Scotland)] and pET11 for expression of his-tagged 14-3-3ε was from Prof. S.O. Døskeland (Department of Biomedicine, University of Bergen). Active p38-regulated/activated protein kinase (PRAK) was from Merck Millipore (Darmstadt, Germany). Unless otherwise stated, reagents were from Sigma-Aldrich (St. Louis, MO, USA).

### Protein expression and purification

Human TH1 was expressed as a (His)_6_-ZZ-hTH1 fusion protein in *E. coli* (BL21 Codon Plus (DE3), Stratagene, CA, US) using the pET-ZZ-1a vector (Kleppe et al. 2014). Expression was induced by 1 mM IPTG, for 4 h, 30 °C. Bacteria were lysed in 50 mM Tris buffer pH 7.5, 300 mM NaCl, 0.5 mg/ml Lysozyme, 1 U/ml Benzonase, Roche protease inhibitor cocktail, 10 mM benzamidine, 1 mM phenylmethyl-sulfonyl-fluoride (PMSF), on ice using French press, and purified using Ni–NTA metal affinity resin (Clontech, CA, US). The fusion tag was removed by proteolytic cleavage overnight on ice, using Tobacco etch virus protease [TeV, 1:50 (mg) TeV:TH] in 15 mM Hepes, pH 7.4, 150 mM NaCl, 1 mM DTT, 5 % glycerol. Samples were then centrifuged (13,000*g*, 10 min) and gel filtrated (Superdex 200 10/300 GL, GE Healthcare, UK) to remove the fusion partner. The preparation was checked by SDS-PAGE for homogeneity. Bovine TH was isolated from adrenal medulla (Haavik et al. 1988). His-tagged 14-3-3ε was expressed in *E. coli* using the pET11 expression vector and purification using Ni–NTA according to manufacturer’s protocol. GST-fused 14-3-3 isoforms (β, γ, η, ζ) were expressed in *E. coli* (BL21-CodonPlusDE3) and purified as described (Kleppe et al. 2014). The uncleaved GST-14-3-3s were used for Surface plasmon resonance measurements. For activity measurements and for dephosphorylation experiments, we used the thrombin cleaved 14-3-3 proteins (20 U/mg fusion protein), with subsequent gel filtration (Superdex 75 10/300 GL, GE Healthcare, UK) in 15 mM Hepes, pH 7.4, 150 mM NaCl. The 14-3-3ε isoform spontaneously forms heterodimers when mixed with the other homodimers of 14-3-3 (Jones et al. 1995; Yang et al. 2006). See supplemental text for details on the preparations of different 14-3-3ε-heterodimers (Fig. S1).

### TH phosphorylation, activity and dephosphorylation

The phosphorylation on Ser19 using PRAK was performed as described (Kleppe et al. 2014); giving phosphorylation stoichiometries between 0.2 and 0.8 mol phosphate/mol TH subunit (TH-pS19). TH activity was measured as described (Toska et al. 2002; Yamauchi et al. 1981). We performed dephosphorylation using lysate from PC12 cells (Leal et al. 2002). The PC12 cells were lysed in 50 mM Hepes, pH 7.4, 1 mM EDTA, 1 mM dithiothreitol (DTT), 0.1 % Triton-X100 and 2× protease inhibitor cocktail (Roche), before centrifugation at 13,000*g* (10 min) and removal of small molecules by three subsequent washes in spin filter microtubes (10 kDa cutoff). Dephosphorylation was performed using 25 mM HEPES (pH 7.2), 130 mM KCl, 1 mM dithiothreitol, 1 mM EGTA, 1x protease inhibitor, 1.25 mg/ml BSA, 5 µM (subunit) phosphorylated TH and 8 µM 14-3-3 subunit, at 25 °C. TH was preincubated for 5 min on ice in the presence or absence of 14-3-3 proteins prior to dephosphorylation (0.4 mg/ml PC12 lysate) and the rate of dephosphorylation was determined at conditions giving less than 20 % turnover. The assay was performed essentially as described by Cohen et al. (Cohen et al. 1988).

### Surface plasmon resonance measurements

The interactions between hTH1 and 14-3-3 isoforms were studied using a Biacore 3000 instrument (GE Healthcare), essentially as described (Kleppe et al. 2014). GST-capture was used for all 14-3-3 dimers except for 14-3-3ε:ε, which was immobilized using direct amine coupling (10 mM Na-acetate, pH 5.4). All binding studies were performed at 25 °C in the standard HBS-P buffer, at flow of 30 μl/min. Sensograms were analyzed using the BIAevaluation v2.2 program by non-linear curve fitting as described (Kleppe et al. 2014).

### Structural analysis

The alignment of human YWHAB, YWHAE, YWHAG, YWHAH and YWHAZ, corresponding to 14-3-3β, ε, γ, η and ζ was performed in ClustalX (v 2.1) using default parameters. The structural analysis of 14-3-3ε (2BR9) was performed using Discovery Studio Visualizer (v 4.1).

## Results and discussion

### Homo- and heterodimers of 14-3-3 bind to TH-pS19 with similar affinities

**Fig. 1:**
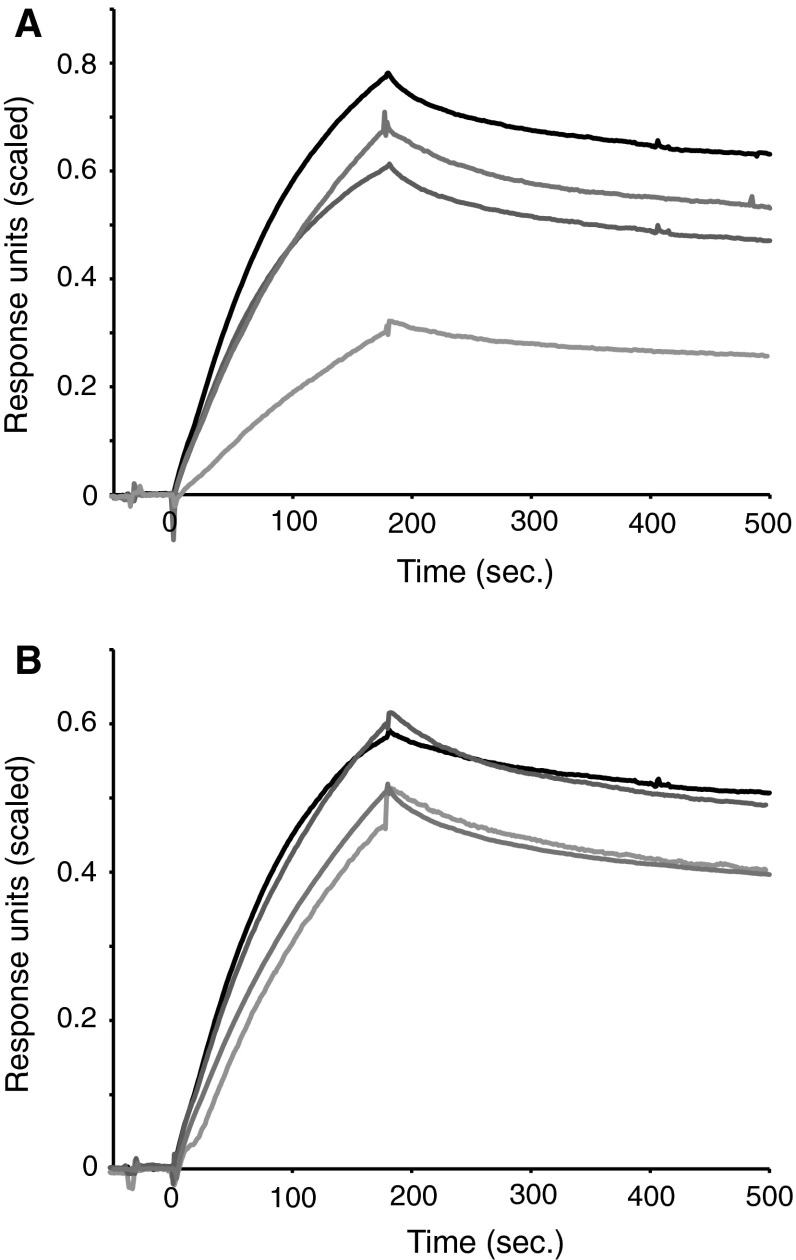
Comparison of binding kinetics of different 14-3-3 dimers to TH-pS19. Binding of TH-pS19 to different immobilized 14-3-3 dimers was measured using Biacore 3000. **a** SPR sensograms showing binding between TH-pS19 (25 nM) and different 14-3-3 homodimers (γ:γ *black*; η:η *red*; β:β *blue*; ζ:ζ *gray*). **b** SPR sensograms showing binding between TH-pS19 (25 nM) and different 14-3-3 ε-heterodimers (ε:γ *black*; ε:η *red*; ε:β *blue*; ε:ζ *gray*). Sensograms were scaled to account for differences in GST-capture levels among the different 14-3-3 dimers and their *R*_max_ value for TH-pS19 binding for a given GST-capture level

Phosphorylation of TH at Ser 19 (TH-pS19) initiates binding to 14-3-3 proteins (Halskau et al. 2009; Itagaki et al. 1999; Kleppe et al. 2014). However, the binding kinetics of different 14-3-3 isoforms, and in particular that of different ε-heterodimers, have not been systematically compared. Using surface plasmon resonance (SPR), we measured the kinetics of Ser19-phosphorylated human TH1 (hTH-pS19) binding to 14-3-3 homo-/heterodimers (Fig. 1a,b). Overall, we found that the different 14-3-3 dimers had very similar *K*_d_-values (1.4–3.8 nM, Table 1). Comparison of the kinetics showed slightly larger differences in the rate constants between the 14-3-3 dimers (maximally 2.4 or 3.1 fold difference between the different *k*_a_-values or the *k*_d_-values, respectively) (Table 1). Only the *k*_d_ rate constants showed significant differences among the 14-3-3 dimers, with 14-3-3γ:γ and β:β having the fastest and slowest dissociation rates, respectively.

**Table 1:** Binding kinetics for the complex between TH-pS19 and different 14-3-3 dimers

14-3-3 dimer	*k*_a_ (10^5^ M^−1^s^−1^)	*k*_d_ (10^−3^ s^−1^)	*K*_d_ (nM)	*t*_0.5_ (Min)
14-3-3ε:ε	2.6 ± 0.90	0.72 ± 0.10	2.8	16
14-3-3γ:γ	3.1 ± 1.5	0.94 ± 0.10	3.0	12
14-3-3ζ:ζ	1.3 ± 0.44	0.38 ± 0.07	2.9	30
14-3-3η:η	2.7 ± 0.80	0.44 ± 0.06	1.6	26
14-3-3β:β	2.2 ± 0.80	0.30 ± 0.02	1.4	38
14-3-3ε:γ	2.0 ± 1.0	0.50 ± 0.07	2.5	23
14-3-3ε:ζ	1.7 ± 0.86	0.65 ± 0.07	3.8	18
14-3-3ε:η	2.9 ± 1.1	0.48 ± 0.04	1.7	24
14-3-3ε:β	2.0 ± 1.0	0.50 ± 0.10	2.5	23

We measured the association and dissociation rate constants for binding of TH-pS19 to 14-3-3 isoforms using SPR (Methods). TH was phosphorylated on Ser19 using PRAK to a stoichiometry of 0.5 phosphates per subunit. Rate constants were fitted as described (Kleppe et al. 2014) and are given ± SEM, estimated by fitting each experiment separately. The equilibrium dissociation constant (*K*_d_) was calculated based on the rate constants (=*k*_d_/*k*_a_) and the dissociation half time (*t*_0.5_) on *k*_d_ (*t*_0.5_ = ln(2)/*k*_d_). Single factor ANOVA of all *k*_a_ and *k*_d_-values showed that only the *k*_d_ constants differed significantly between the 14-3-3 dimer types (*P* = 8.5 10^−5^)

It could be speculated if such moderate differences in rate constants could lead to preferential accumulation of TH:14-3-3 complexes containing certain 14-3-3 dimers. However, this would depend on the signaling dynamics of the cell as well as the cellular availability of the different 14-3-3 dimer types. Cell studies have suggested a preferential interaction of TH with 14-3-3ζ over η (Wang et al. 2009). Here, we show that this is unlikely to be caused by differences in binding kinetics. Possibly, the 14-3-3 selectivity reported in cell studies are due to different cellular localization or availability of the two 14-3-3 proteins. Also, the 14-3-3η isoform has been reported to regulate TH stability in PC12 cells (Nakashima et al. 2007). Interestingly, 14-3-3η is also reported to interact with the E3-ubiquitin ligase Parkin, and regulated by α-synuclein (Sato et al. 2006). These findings suggest a more complex relationship between 14-3-3 binding and its regulation. Thus, the observed complexes formed and the functional outcome could be a result of competitive interactions between different 14-3-3 target proteins and, at least for TH, different 14-3-3 dimer types.

### Differential activation of TH by 14-3-3 isoforms

The 14-3-3 proteins are known as phosphorylation dependent activators of TH and tryptophan hydroxylase (TPH) (Itagaki et al. 1999; Toska et al. 2002; Yamauchi et al. 1981). However, conflicting results have been reported on their potency to activate TH (summarized in Table S1) (Haycock and Wakade 1992; Sutherland et al. 1993; Toska et al. 2002). We therefore compared the ability of different 14-3-3 isoforms and heterodimers to modulate the activity of bovine and human TH (bTH and hTH), isolated from native and recombinant sources, respectively. Different assay conditions have been used in earlier reports to measure 14-3-3 mediated TH activation. As it is known that the regulatory properties of TH are different in the presence of the natural cofactor 6R-tetrahydrobiopterin (BH_4_) compared to the synthetic analogue 6-methyltetrahydropterin (6MPH_4_) used in early studies (Yamauchi et al. 1981), we first performed a systematic comparison of TH activation using both 6MPH_4_ and BH_4_. The two cofactors gave similar activation of either bTH or hTH1 phosphorylated by PRAK, both for the mammalian 14-3-3 isoforms and for yeast 14-3-3 (BMH1). As we have previously shown, 14-3-3 binding has no effect on the *K*_m_ of TH for BH_4_ (Toska et al. 2002). Also, we did not find a significant effect of 14-3-3 on the *K*_m_ or substrate inhibition constant (*K*_si_) of TH for its substrate tyrosine.

**Fig. 2:**
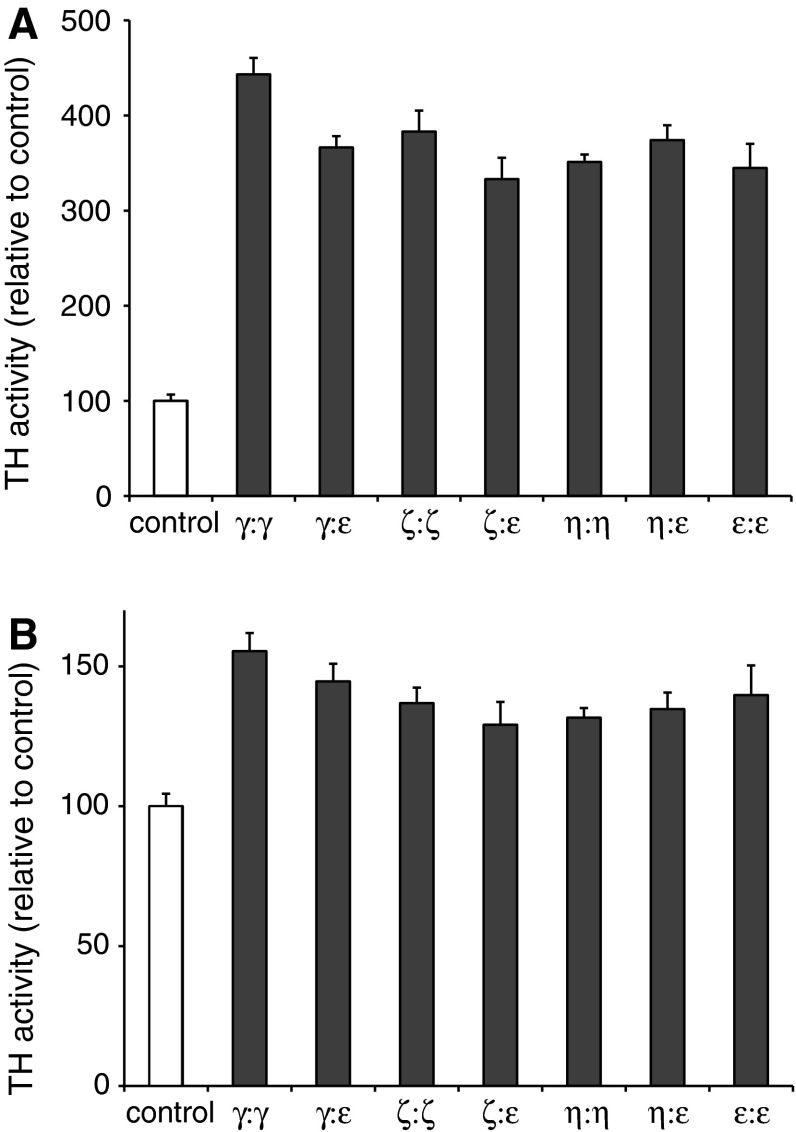
Activation of TH by different 14-3-3 isoforms. TH phosphorylated by PRAK (TH-pS19) was assayed for activity in the presence or absence of different 14-3-3 isoform combinations (12.5–100 µg/ml) to yield a 14-3-3 activation response curve. The maximal TH activation is reported here. Panel **a** shows the relative activation of bovine TH (PRAK phosphorylated) by different homo- and heterodimers of 14-3-3. All were significantly higher than the control (bTH-pS19 without 14-3-3) (*P* < 1.0 10^−5^), with 14-3-3γ:γ as the most potent activator (*P* < 0.005). For all 14-3-3ε-heterodimers, except that of 14-3-3η:η, the activation was slightly, but significantly lower compared to that of their non-ε-homodimer (*P* < 0.05). Panel **b** shows the relative activation of hTH1 (PRAK phosphorylated) by different homo- and heterodimers of 14-3-3. All were significantly higher than the control (*P* < 0.0013), but less potent than 14-3-3γ:γ (*P* < 0.015, 14-3-3ε:ε not significant). The 14-3-3ε-heterodimer of γ (*P* < 0.008) showed less potent activation of TH compared to its homodimeric form. All *P* values were calculated using the two-tailed *t* test and corrected for multiple comparisons

Using BH_4_ as the cofactor we investigated, in more detail, possible differences between the various 14-3-3 dimer types in their activation of bTH and hTH1 (Fig. 2). The activation of non-phosphorylated TH by different 14-3-3 proteins was <14 % and that of BSA (50 µg/ml) on both TH and PRAK phosphorylated TH was <10 % (data not shown). However, we observed a strong activation (*t* test, *P* < 10^−5^) of bTH-pS19 ranging from 3.3- to 4.4-fold in the presence of different 14-3-3 dimers (Fig. 2a). Thus, the homodimer of 14-3-3γ gave the highest activation (*P* < 0.005; 14-3-3γ:γ different from other 14-3-3dimers), whereas the 14-3-3ζ:ε heterodimer gave the lowest activation. There was a slight, but significant difference between the homodimers in their activation of bTH-pS19, compared to their respective 14-3-3ε-heterodimer (*P* < 0.05). For the hTH-pS19 protein, we observed a similar, but much less pronounced activation by the 14-3-3 proteins (Fig. 2b). Thus, 29–55 % activation was observed (*P* < 1.3 × 10^−3^), with 14-3-3γ homodimer and 14-3-3ζ:ε as the most and least potent activators, respectively.

The bTH from native sources was clearly activated more strongly by 14-3-3 proteins than hTH produced in *E. coli*. We therefore tested if possible co-purified components in the bTH preparation could potentiate the activation of hTH. However, we found no additional activation of the hTH by 14-3-3 proteins by mixing with either non-phosphorylated bTH or PRAK phosphorylated bTH. Thus, we suspect that the variable activation by 14-3-3s could be due to alterations in the state of the bTH protein itself, such as additional covalent or non-covalent protein modifications, oxidation or catecholamine binding. In line with this, we noted that the majority of studies reporting strong activation of TH by 14-3-3 proteins generally used TH prepared from native sources, including SF21 insect cells (Itagaki et al. 1999; Tanji et al. 1994; Yamauchi et al. 1981). Additionally, in intact systems, a differential effect of the 14-3-3 dimers on TH stability or turnover cannot be excluded (Nakashima et al. 2007; Nakashima et al. 2011).

### The impact of 14-3-3 proteins on the dephosphorylation of TH

**Fig. 3:**
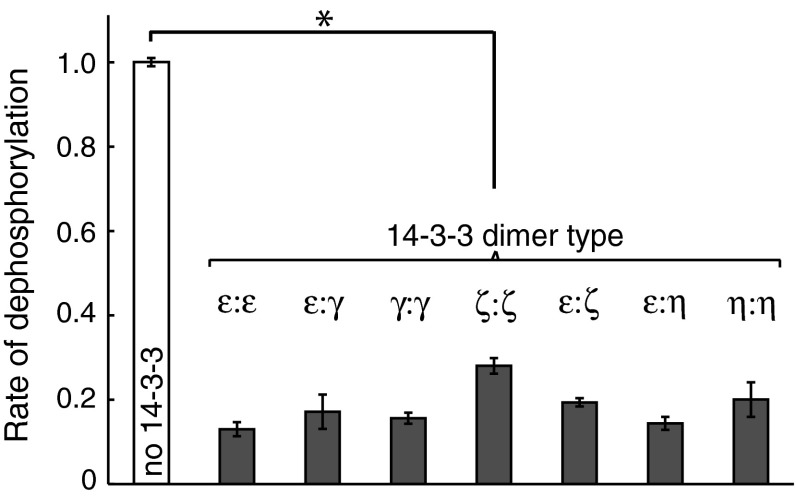
Dephosphorylation of TH in presence and absence of different 14-3-3 s. The assay was performed at 25 °C for 5–10 min incubating 10 µM 14-3-3 and 5 µM of phosphorylated TH with 0.4 mg/ml PC-12 cell lysate as described in Methods. Dephosphorylation of TH-pS19 (0.15 stoichiometry) was measured in presence and absence of different 14-3-3 homo/hetero dimers. The error bars show the st.dev. from three different experiments (*P < 0.001, two-tailed *t* test)

Major TH Ser/Thr phosphatases include Protein phosphatase 2A (PP2A) and 2C (PP2C) (Bevilaqua et al. 2003; Haavik et al. 1989; Leal et al. 2002). To mimic dephosphorylation of TH in cells, we used cell lysate from PC12 cells that contain intact and relevant protein complexes of these protein phosphatases. Such lysates have been used previously to assess TH dephosphorylation (Bevilaqua et al. 2003) and were used here to investigate possible differences in the dephosphorylation of human TH-pS19 in the presence of different mammalian 14-3-3 homo- and heterodimers. All tested 14-3-3 homo/heterodimers strongly inhibited dephosphorylation of Ser19 (72–87 %) (Fig. 3). We have previously described the maximal binding of two 14-3-3γ dimers for each TH-pS19 tetramer (Kleppe et al. 2014). We also observed a mono-exponential dephosphorylation rate of TH-pS19 in the presence of 14-3-3γ suggesting that all four sites are protected in the complex. Here, we observed a strong inhibitory effect of all the different 14-3-3 dimers on TH-pS19 suggesting similar modes of binding for the 14-3-3 proteins.

However, the inhibition appeared to be slightly different between the 14-3-3 dimers, 14-3-3ε:ε having the strongest inhibition and 14-3-3ζ:ζ having the weakest. Thus, the observed potency of dephosphorylation inhibition did not correspond with the rate of dissociation of the complex as measured by SPR. Theoretically, such effects can arise from different conformational states in the complexes and from multiple reaction steps in the association and dissociation process. We have previously observed such rate differences between the *k*_d_-value measured using SPR and the dephosphorylation at high levels of phosphatase (where TH-pS19 was 50 % dephosphorylated <5 s. in absence of 14-3-3) (Kleppe et al. 2014). However, injection of high levels of phosphatase during dissociation in the SPR measurements gave a rate constant corresponding to the one measured in solution, suggesting multiple states for the TH:14-3-3 complexes. However, a detailed kinetic investigation of this phenomenon was outside the scope of this study.

### Structural variation in the surface of the 14-3-3 isotypes

Few examples exist on comparable affinity analysis of different 14-3-3 dimers binding to the same target protein. The literature reports both selective and non-selective binding of 14-3-3 isoforms to target proteins, suggesting that the target proteins gain affinity by interacting with non-conserved or conserved surface areas of 14-3-3 proteins, respectively, in addition to the phospho-Ser/Thr interaction site (Subramanian et al. 2001; Vincenz and Dixit 1996).

**Fig. 4:**
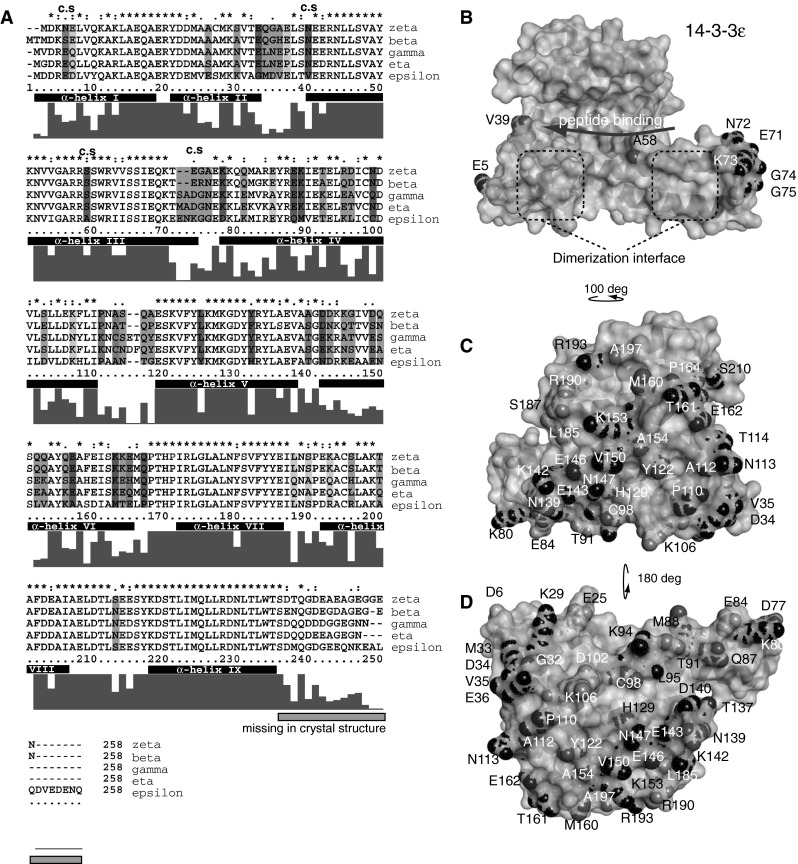
Differences between surface-exposed amino acids of the 14-3-3 isoforms studied. Panel **a** shows the alignment of human 14-3-3ζ, β, γ, η and ε, where surface-exposed amino acid residues that differ between the isoforms are shaded in color coding; *blue* (14-3-3ζ and/or β differ from the rest), *red* (14-3-3ε different than the rest), *green* (14-3-3γ and/or η different), *gray* (differences between all groups) and orange (14-3-3ε and γ/η or ζ/β differ). The color coding is used correspondingly in the panels showing the surface structure of 14-3-3 (**b**–**d**). The amino acids that are part of α-helix I to IX are marked in the alignment. Panels **b**–**d** show the surface area of a subunit of human 14-3-3ε (2BR9). *Gray* surface is conserved between 14-3-3ζ, β, γ, η and ε, whereas colored surface mark differences [according to color code in (**a**)]. Panel **b** shows the conserved binding groove of 14-3-3ε, with the dimerization interface exposed and the peptide binding site illustrated with an *arrow*. The colored amino acids that contribute to isoform variation of the conserved binding groove of 14-3-3 are marked by c.s (conserved surface) in the alignment. Panels **c**, **d** show the surface of 14-3-3ε obtained by rotating the view in panel (**b**) as illustrated. These surfaces correspond to the variable surface of the outer wall (**c**) and the outer floor (**d**) of the 14-3-3 U-shaped structure. The amino acid numbering is according to residue numbers in 14-3-3ε

Analysis of the surface-exposed amino acids in the different 14-3-3 isoforms studied here (Fig. 4) suggests that selectivity of targets binding inside the binding groove of 14-3-3 would mainly arise from interactions with the last amino acids of α-helix III (H-III) and the loop between H-III and H-IV (Fig. 4a, b). Interactions with surface areas on the outer side of the wall (H-V–H-IX) and the outer surface of the floor (H-I–H-IV) of the binding groove would provide opportunities for selectivity over the whole surface area (Fig. 4c, d). Patches of red, green and blue, corresponding to amino acid differences in 14-3-3ε, γ/η and ζ/β, respectively, suggest that selectivity between these groups, but rarely within, can be expected. The analysis also readily illustrates some of the unique features of 14-3-3ε.

Our data suggest that the TH-pS19:14-3-3 interaction is driven mainly by a surface interface that is conserved among the tested 14-3-3 isoforms. This would translate to the inner side of the binding groove, including the top of the wall (H-IX). Interactions with H-IX would provide close spatial vicinity to the acidic C-terminal of 14-3-3, which is a region without any structural resolution and of high amino acid diversity. Its acidity, viewed in context with the known activation of TH by heparin and the reported inhibition of 14-3-3 binding by heparin, provides a plausible mechanism of TH activation by 14-3-3 proteins, which was somewhat different between the isoforms. The possible impact of 14-3-3 C-terminal in TH binding and activation, remains however, to be confirmed experimentally.

In the reported 14-3-3ζ:Serotonin N-acetyltransferase structure, there is evidence for extensive interactions within the binding groove of 14-3-3, where 34 of the 37 amino acids that participate in the protein–protein interactions are conserved between the 14-3-3 isoforms (Obsil et al. 2001). Although not directly compared, less 14-3-3 isoform selectivity would therefore be expected for serotonin N-acetyltransferase. The 14-3-3 target proteins RGS3 and phosducin show interactions outside the binding groove, in a less conserved area of 14-3-3 (Kacirova et al. 2015; Rezabkova et al. 2011). For neither RGS3 nor phosducin the binding affinities to the different 14-3-3 isoforms have been compared, although RGS3 is reported to interact with 14-3-3β and τ in addition to 14-3-3ζ (Benzing et al. 2000; Niu et al. 2002; Rezabkova et al. 2011). Of the mapped interaction surface between 14-3-3 and RGS3, H-VI and H-VIII are areas that can give rise to isoform selectivity that would fit with its interaction with 14-3-3β, ζ and τ. This still needs to be experimentally tested, e.g. by mutagenesis, if it is in fact so, and whether such selectivity can be extended to a biological functional level. Still, the question about functional differences between 14-3-3 isoforms is one of the major unresolved issues in the biology of these proteins.

## Conclusions

We have shown a robust interaction of TH-pS19 across multiple 14-3-3 dimer forms expected to be present in dopaminergic cells. This conserved interaction was reflected in a consistent activation of TH and inhibition of TH-dephosphorylation with moderate 14-3-3 isotype preferences. Thus, we suggest the presence of different categories of 14-3-3 targets; those that convey isotype selectivity by large surface interactions on the outer surface of the phospho-Ser/Thr peptide binding channel, whereas others that interact non-selectively as the binding interface covers areas within the binding groove and the top of the wall (C-terminal H-IX). Access to such structural information will also be important for selective pharmacological targeting of 14-3-3 protein interactions.

## Electronic supplementary material

Below is the link to the electronic supplementary material.

Supplementary material 1 (PDF 544 kb)

## Abbreviations

BH_4_: Tetrahydrobiopterin
PRAK: P38-regulated/activated protein kinase
SPR: Surface plasmon resonance
TH: Tyrosine hydroxylase
TH-pS19: TH phosphorylated on Ser19
bTH: Bovine TH
hTH: Human TH1
