# Supplementary material for: Regulation of tyrosine hydroxylase is preserved across different homo- and heterodimeric 14-3-3 proteins
Source: Amino Acids. 2016 Jan 29;48:1221–9. doi: 10.1007/s00726-015-2157-0 (PMC4833811; doi:10.1007/s00726-015-2157-0)
Supplement: Supplementary file 1 — Supplementary material 1 (PDF 544 kb) [file 726_2015_2157_MOESM1_ESM.pdf]

Supplemental text

The heterodimers of His-14-3-3ε with 14-3-3β, ζ, γ and η were generated from the isolated pure homodimers (GST-14-3-3 or cleaved 14-3-3 fusion proteins) by incubating them in a 1:1.5 molar ratio (14-3-3ε:14-3-3γ, η or ζ) over night (4 °C) in HEPES buffer supplemented with DTT (1 mM). The protein mix containing His-14-3-3ε-heterodimers and residual 14-3-3γ:γ, 14-3-3ζ:ζ, 14-3-3η:η or 14-3-3β:β-homodimers were passed through an equilibrated Ni-NTA column to remove the 14-3-3γ/ζ/η/β homodimers. The 14-3-3ε-heterodimers were eluted by phosphate buffer containing 250 mM imidazole, with subsequent gelfiltration on a Superdex 200 (HR 10/30) column (15 mM HEPES, 150 mM NaCl) before storage in liquid nitrogen. All preparations were checked by SDS-PAGE for homogeneity (Fig. S1).

Supplemental Figure

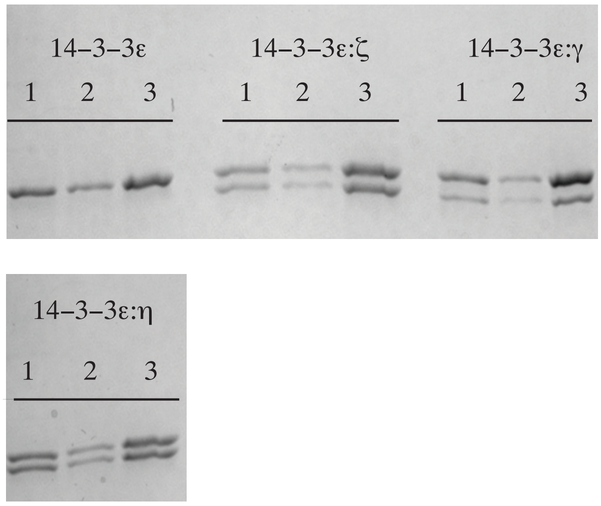

Fig. S1. SDS-PAGE of pulled down heterodimers. The panel shows prepared pure His-14-3-3ε heterodimers. To evaluate the heterodimerization on our samples, we pulled down prepared heterodimers by Ni-NTA agarose, and the samples from the pulled down protein and the supernatant was compared to the original sample by evaluation using SDS PAGE. The

gels show preparations of His-14-3-3ε:ε, His-14-3-3ε:ζ. His-14-3-3ε:γ, and His-14-3-3ε:η. For all of them the following setup was used: Lane 1) Prepared 14-3-3 heterodimer. Lane 2) Supernatant. Lane 3) Eluate from Ni-NTA agarose.

Supplemental Table

Table S1. Literature reported effects of 14-3-3 proteins on TH activity.

Study	TH source	14-3-3 source	Kinase/ stimulation	Buffer/Suppliments	Activation
(1)	Bovine adrenal medulla	Cerebral crude extract	CaMKII from rat cerebral cortex	Mes (pH 6.5), EDTA, $6MPH_4$	146 %
(2)	Bovine adrenal medulla	Bovine forebrain	CaMKII from rat brain	Tris-maleat (pH 6.4), $6MPH_4$	150-180 %
(3)	Recombinant human TH from SF21 cells	Recombinant rat GST-14-3-3β & bovine 14-3-3η	CaMKII from rat brain	Mes (pH 6.5), $6MPH_4$, Fe-added	225 %
(4)	Recombinant human TH1 from *E. coli*	Recombinant BMH1	PRAK	HEPES (pH 7.0), BH_4, Fe-added	260 %
(5)	Recombinant human TH1 from *E. coli*	Recombinant rat 14-3-3γ, ζ, and η	PRAK	HEPES (pH 7.0), BH_4, Fe-added	γ: 115%<br>ζ: 146%
(6)	Recombinant human TH from *E. coli*	Sheep brain	CaMKll & MK2	HEPES (pH 7.0), BH_4, Fe-added	no
(7)	Rat perfused adrenal gland	Bovine brain	Nicotine & Muscarine	Tris-phosphate (pH 7.2), BH_4	no

1. T. Yamauchi, H. Nakata, H. Fujisawa, A new activator protein that activates tryptophan 5-monooxygenase and tyrosine 3-monooxygenase in the presence of Ca2+-, calmodulin-dependent protein kinase Purification and characterization, Journal of Biological Chemistry, 256 (1981) 5404–5409.
2. M. Tanji, R. Horwitz, G. Rosenfeld, J. Waymire, Activation of Protein Kinase C by Purified Bovine Brain 14-3-3: Comparison with Tyrosine Hydroxylase Activation, Journal of Neurochemistry, 63 (1994) 1908–1916.
3. C. Itagaki, T. Isobe, M. Taoka, T. Natsume, N. Nomura, T. Horigome, S. Omata, H. Ichinose, T. Nagatsu, L.A. Greene, others, Stimulus-coupled interaction of tyrosine hydroxylase with 14-3-3 proteins, Biochemistry, 38 (1999) 15673–15680.
4. K. Toska, R. Kleppe, C.G. Armstrong, N.A. Morrice, P. Cohen, J. Haavik, Regulation of tyrosine hydroxylase by stress-activated protein kinases, Journal of Neurochemistry, 83 (2002) 775–783.
5. O. Halskau, M. Ying, A. Baumann, R. Kleppe, D. Rodriguez-Larrea, B. Almås, J. Haavik, A. Martinez, Three-way interaction between 14-3-3 proteins, the N-terminal region of tyrosine hydroxylase, and negatively charged membranes, Journal of Biological Chemistry, 284 (2009) 32758–32769.
6. C. Sutherland, J. Alterio, D.G. Campbell, B. Bourdelles, J. Mallet, J. Haavik, P. Cohen, Phosphorylation and activation of human tyrosine hydroxylase in vitro by mitogen-activated protein (MAP) kinase and MAP-kinase-activated kinases 1 and 2, European Journal of Biochemistry, 217 (1993) 715–722.
7. J.W. Haycock, A.R. Wakade, Activation and Multiple-Site Phosphorylation of Tyrosine Hydroxylase in Perfused Rat Adrenal Glands, Journal of Neurochemistry, 58 (1992) 57–64.
